# Revisiting the combined approach of Yaşargil for microsurgical removal of intra-extraventricular and pure intraventricular craniopharyngiomas

**DOI:** 10.1007/s00701-025-06560-1

**Published:** 2025-05-23

**Authors:** Oğuz Kağan Demirtaş, Abuzer Güngör, Yücel Doğruel, Fahrettin Kelestimur, Hatice Türe, Uğur Türe

**Affiliations:** 1 Department of Neurosurgery, Yeditepe University School of Medicine, İstanbul, Türkiye; 2 Department of Neurosurgery, Etlik City Hospital, Ankara, Türkiye; 3 Department of Neurosurgery, Faculty of Medicine, İstinye University, İstanbul, Türkiye; 4 Department of Neurosurgery, Health Sciences University, Tepecik Training and Research Hospital, İzmir, Türkiye; 5 Department of Endocrinology, Yeditepe University School of Medicine, İstanbul, Türkiye; 6 Department of Anesthesiology, Yeditepe University School of Medicine, İstanbul, Türkiye

**Keywords:** Combined approach, Craniopharyngioma, Microsurgical resection, Transcallosal transforaminal approach, Pterional approach

## Abstract

**Objective:**

Craniopharyngiomas originate from squamous epithelium in the pituitary stalk, tend to expand into surrounding tissues, and have high recurrence rates when residual tumor remains. Therefore, gross total resection should be the goal at initial surgery. Yaşargil described the combined approach, involving both anterior interhemispheric transcallosal transforaminal and pterional transsylvian approaches in the same session for intra-extraventricular and pure intraventricular craniopharyngiomas. This study presents our series, the first since Yaşargil’s publications, of patients operated on with this approach.

**Methods:**

Data were prospectively collected (September 2006–August 2024) from patients undergoing endoscope-assisted combined craniopharyngioma surgery. First, parasagittal craniotomy was performed, and the tumor was resected microsurgically via the anterior interhemispheric transcallosal transforaminal route. Then, the residual tumor in the parachiasmatic area was removed via the pterional craniotomy and transsylvian route. Since January 2018, intraoperative MRI has been used to confirm gross total resection.

**Results:**

During the study period, 67 craniopharyngioma patients underwent surgery, and combined approach was performed in 12 cases. Gross total resection was achieved in 11 of the 12 patients who underwent the combined approach, while one had a near-total resection. The patient who underwent near-total resection had a history of two prior surgeries and radiotherapy and was the only case of recurrence (mean follow-up: 97 months). The stalk was resected in all patients and hormone replacement therapy was required.

**Conclusion:**

The basic principle in the treatment of craniopharyngiomas, which have high recurrence rates in the presence of residual tumor and locally aggressive behavior, is gross total resection in the initial surgery. Gross total resection offers the opportunity for cure and is critical in the course of the disease. The combined approach is an effective and safe technique to achieve gross total resection in the initial surgery for patients with intra-extraventricular and pure intraventricular craniopharyngiomas.

**Supplementary Information:**

The online version contains supplementary material available at 10.1007/s00701-025-06560-1.

## Introductıon

Craniopharyngiomas are tumors derived from the remains of squamous cells along the pituitary stalk. They were first named pituitary epithelial tumors by Jakob Erdheim, then later called craniopharyngiomas by Harvey Cushing [35]. Craniopharyngiomas tend to expand extensively into the surrounding tissues but preserve the arachnoid plane [44]. The fundamental philosophy in treating patients with craniopharyngiomas is gross total resection (GTR) during the inital surgery, which provides maximal recurrence-free survival [43–45]. However, surgery for a craniopharyngioma is still challenging because of the close proximity to vital structures such as the hypothalamus, optic system, and branches of the internal carotid artery (ICA) and the basilar artery. A combination of the anterior interhemispheric transcallosal transforaminal approach (AITT) and the pterional transsylvian approach was described by Yaşargil for GTR of intra-extraventricular and pure intraventricular craniopharyngiomas [40, 43–45].

The aim of this study was to share our series of patients operated on with this combined approach. We hope to revive this approach to give it the attention it deserves since Yaşargil’s publications [43–45].

## Methods

This study was carried out by retrospectively analyzing prospectively collected data of patients with a pathological diagnosis of craniopharyngioma who underwent surgery in the Department of Neurosurgery at Yeditepe University from September 2006 through August 2024. All participants provided informed consent, and the study was conducted in accordance with the principles outlined in the Helsinki Declaration, ensuring ethical standards were adhered to throughout the research process.

**Fig. 1:**
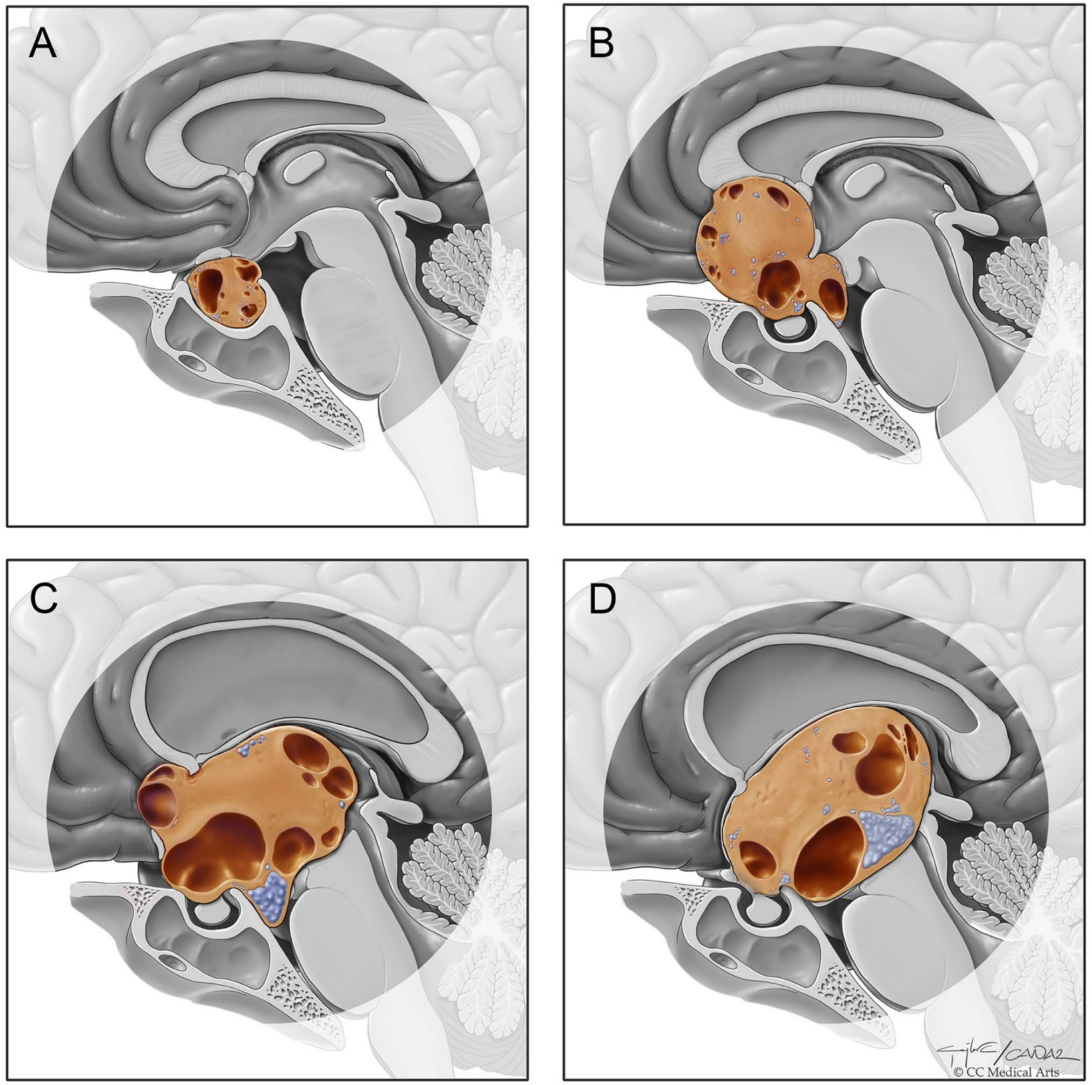
Artistic illustration of the anatomical subtypes of craniopharyngiomas **A**) intrasellar, **B**) parachiasmatic, **C**) intra-extraventricular, **D**) pure intraventricular

**Fig. 2:**
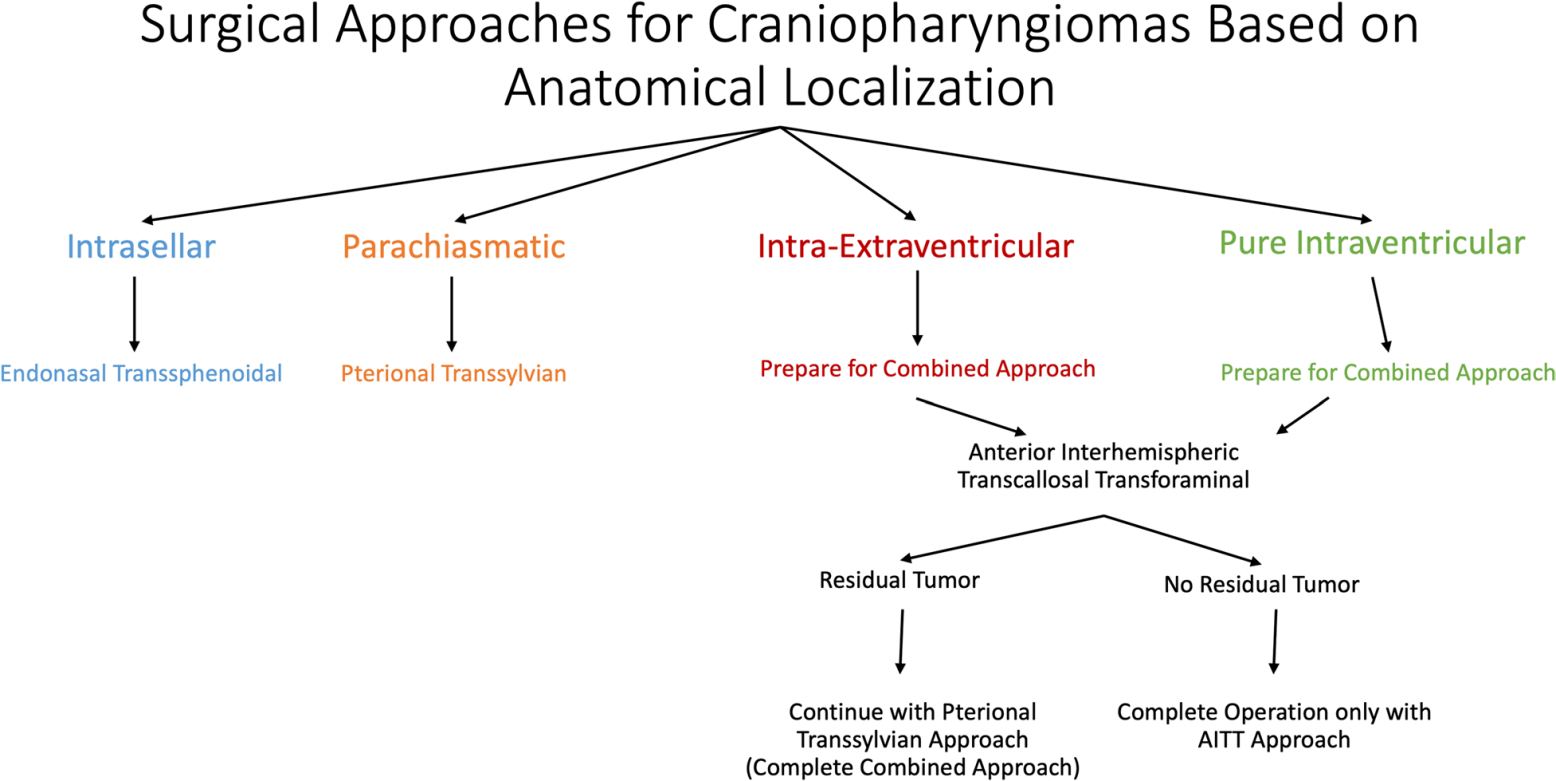
Surgical approaches for craniopharyngiomas based on anatomical localization. The endonasal transsphenoidal approach was preferred for intrasellar tumors. For parachiasmatic tumors, the pterional transsylvian approach was used. The combined approach was planned for intra-extraventricular and pure intraventricular tumors. First, a transcallosal approach is performed. The operation is terminated after this approach if gross total resection is achieved. In the presence of residual tumor, the operation is combined with a pterional transsylvian approach

To determine the surgical approach, we classified craniopharyngiomas into four groups according to anatomical location: intrasellar, parachiasmatic, intra-extraventricular, and pure intraventricular (Fig. 1). The endonasal transsphenoidal approach was preferred for intrasellar tumors. The pterional approach was preferred for parachiasmatic tumors. In patients with intra-extraventricular or pure intraventricular lesions, surgery was planned with the combined approach. After the AITT approach, the patient was evaulated for the existence of residual tumor. If GTR was achieved, the operation was terminated. If residual tumor remained, the pterional approach was added to achieve GTR (Fig. 2).

Patients with intra-extraventricular and pure intraventricular tumors who underwent the combined approach were included in this study.

Data collected for each patient included demographic information, main symptoms, results of the preoperative neurological and preoperative/postoperative endocrinological evaluations, the degree of surgical resection, preoperative and postoperative magnetic resonance images (MRI), results of vision field tests, peri-operative complications, pathological diagnoses, postoperative endocrinological and biochemical parameters, and clinical histories from follow-up exams.

### Surgical technique

Just before surgery, patients are administered hydrocortisone at an appropriate dose to protect them from the stress of postoperative cortisone withdrawal. Prophylactic antibiotics and antiepileptics are also administered before surgery. Intravenous fluid is limited as much as possible during surgery. During the operation, hypotension is avoided; normotension is the goal.

**Fig. 3:**
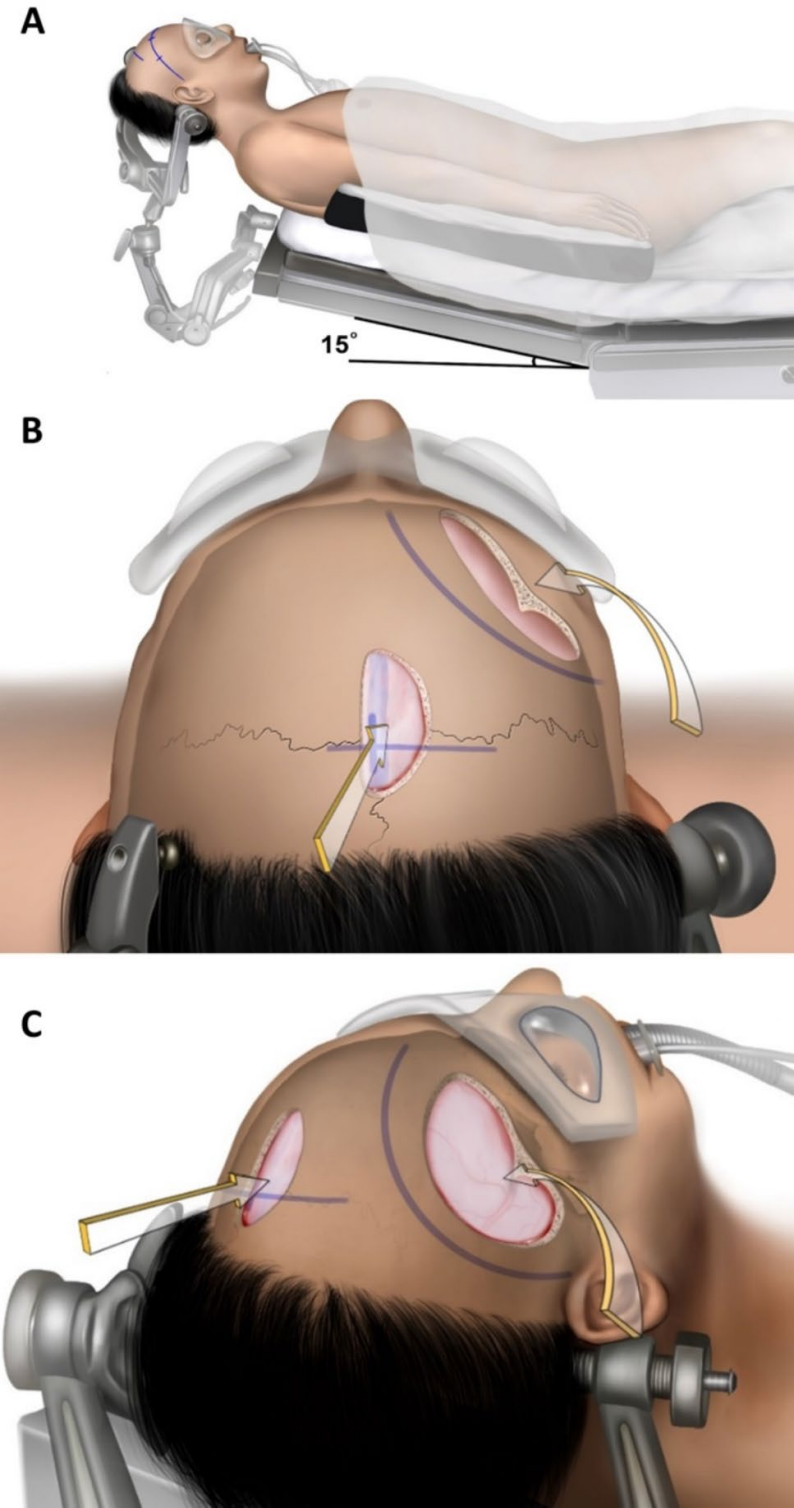
Surgical position and skin incisions. **A** First, for the interhemispheric approach, the head is fixed in the supine position with neutral, slight flexion. **B** After a linear skin incision is made parallel to the coronal suture, a parasagittal craniotomy is performed. **C** After the first approach is completed, if residual tumor remains, surgery is continued with a pterional approach. The headholder is loosened under sterile drapes and re-positioned through rotation and a tilt of approximately 30 degrees to the opposite side. After repositioning, the pterional approach is carried out

When the patient is draped, both the parasagittal and pterional fields are covered for a combined approach. The head is positioned in a neutral, slightly flexed position suitable for the AITT approach. If necessary, the head is repositioned by loosened the Mayfield® head-holder to allow access for the pterional approach without compromising the sterile field. Different skin incisions are planned for both craniotomies so that the incision is not unnecessarily lengthened when the tumor is completely removed through the transcallosal approach (Fig. 3).

#### Anterior ınterhemispheric transcallosal transforaminal approach

Magnetic resonance venography is used to determine the craniotomy site based on the superficial venous structures. If the venous anatomy is suitable, an opening more posterior than the classic right parasagittal craniotomy is made to access tumor areas beneath the anterior commissure. Parasagittal bridging veins should be mobilized through arachnoid dissection and none should be sacrificed. Moist cottonnoid can be placed anterior and posterior to the dissection site for a more gentle retraction. We do not prefer to use a fixed retractor.

Arachnoid dissection is deepened to the level of the corpus callosum and cottonoid-guided intraoperative ultrasound is used to determine the optimal site for a callosotomy [26]. The callosotomy, foramen of Monro, and tumor route are checked with ultrasound. The callosotomy site is adjusted so that the foramen of Monro and the infundibular region are in a single plane along one axis. An approximately 5-mm callosotomy is done between the pericallosal arteries on the corpus callosum. The callosotomy allows the outflow of cerebrospinal fluid (CSF) from the lateral ventricle. The opposite lateral ventricle, which is not drained, may bulge with pressure and block the surgeon’s view. In this case, CSF outflow from the opposite lateral ventricle should be ensured through fenestration of the septum pellucidum, thereby allowing the surgeon to observe both foramina of Monro.

The volume of solid tumors is reduced primarily by internal debulking. Then, separation from the ventricular walls is attempted. With this approach, hypothalamic adhesions can be dissected safely and the portion of the tumor within the ventricle and around the stalk can be completely resected. The residual tumor is directly observed through microscopic inspection or endoscopic visualization, the operation is continued with a combined approach. If no residual tumor is detected during inspection, the operation is concluded, and since January 2018, this is further confirmed with intraoperative MRI.

#### Pterional transslyvian approach

For this approach, the patient’s head is rotated and tilted 30 degrees to the opposite side by loosened and refixing the Mayfield headholder under sterile covers. The right side is used for the pterional approach. After the classic pterional craniotomy, the sphenoid ridge is drilled with a high-speed drill to ensure adequate exposure. The dura is opened in semicircular fashion and retracted towards the sphenoid wing. The proximal Sylvian fissure is completely dissected and the optic nerve, ICA, middle cerebral artery, and anterior cerebral artery are disclosed. The dural fold surrounding the optic nerve is cut before tumor manipulation to prevent injury to the optic nerve. The anterior clinoidectomy is performed intradurally so that a wider exposure is obtained and the ICA is mobilized more easily, if necessary. If there is a sinus in the anterior clinoid (pneumatized), a clinoidectomy is avoided because of the possibility of a CSF leak.

**Fig. 4:**
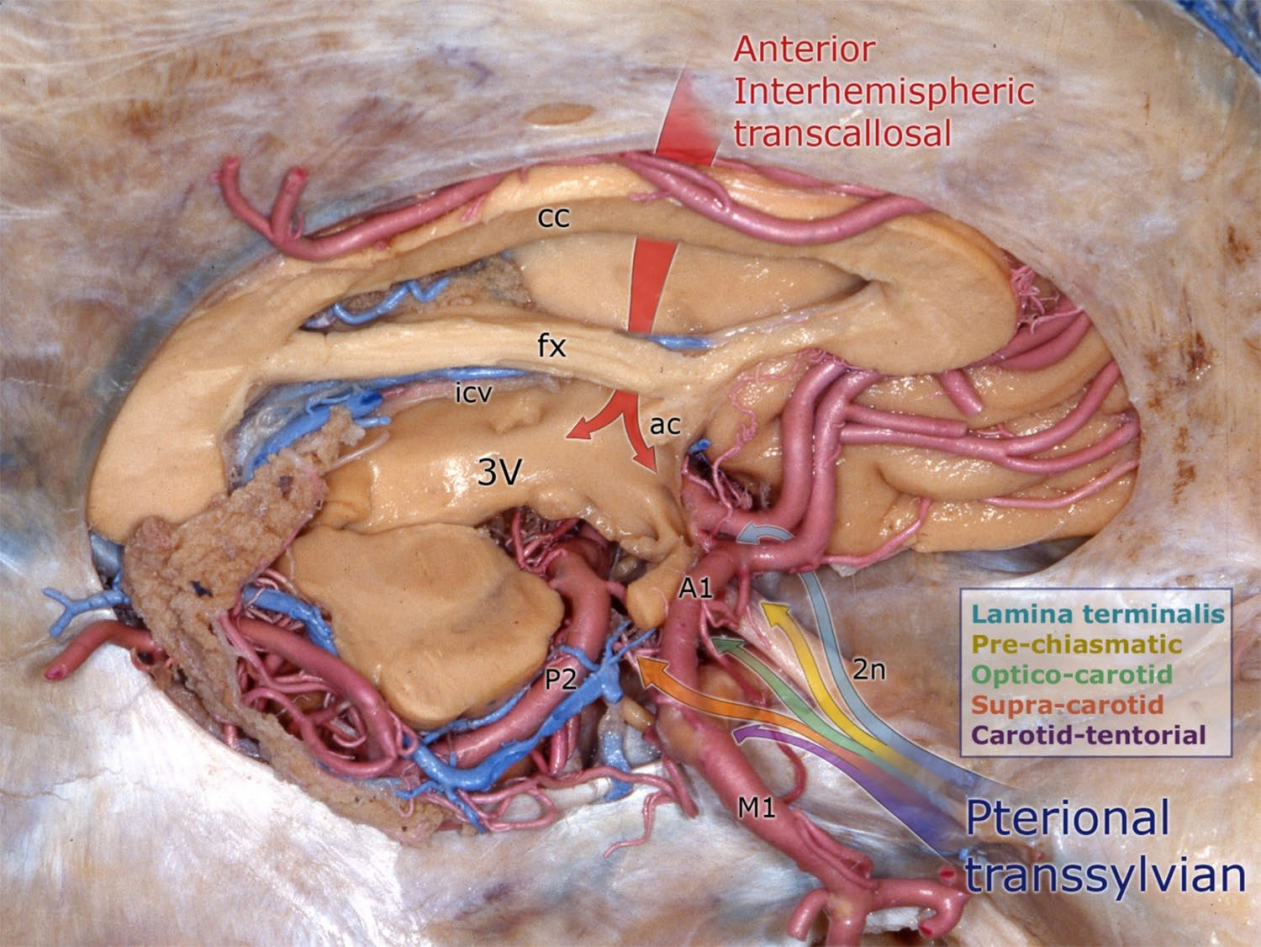
Demonstration of the combined approach on an anatomical specimen. Red arrow: interhemispheric transcallosal route. The multicolor arrow indicates the pterional transsylvian route with optico-carotid, prechiasmatic, supracarotid, and carotid-tentorial intervals. Red: transcallosal; blue: lamina terminalis; yellow: prechiasmatic; green: optico-carotid; orange: supracarotid; purple: carotid, tentorial; 2n: optic nerve; 3 V: third ventricle; A1: pre-communicating segment of the anterior cerebral artery; ac: anterior commissure; cc: corpus callosum; fx: fornix; icv: internal cerebral vein; M1: horizontal segment of the middle cerebral artery; P2: post-communicating segment of the posterior cerebral artery

According to the relationship of the tumor to important structures such as the optic nerve, chiasm, and the ICA and its branches, the tumor is removed from the optico-carotid, prechiasmatic, supracarotid, carotid-tentorial, and lamina terminalis intervals (Fig. 4). The pterional route offers the opportunity to work through many triangles. In most cases, using two or three of these is usually possible and sufficient. During debulking, the tumor stalk is cut as soon as it is identified, allowing the tumor to be freed and facilitating its manipulation. Intraoperative desmopressin is administered after the stalk is cut. As this tumor is interarachnoid, once the arachnoid plane is identified, it is possible to separate the tumor from the surrounding neurovascular structures, thus achieving GTR without neurovascular injury.

Craniopharyngiomas receive their blood supply from the branches of the ICA, such as the superior hypophyseal artery. They do not receive perforating branches from the posterior communicating artery or the basilar artery. Therefore, when staying within the arachnoid plane, it is possible to dissect the tumor away from the posterior neurovascular structures. Bipolar coagulation is avoided as much as possible during resection, as a clear separation of tumor feeders and perforators of surrounding neural structures is not always possible. Some branches of the superior hypophyseal artery supply the chiasma while others supply the tumor. Until these can be clearly differentiated, the surgeon should avoid coagulating these branches.

The operation is terminated once no residual tumor is seen and bleeding stops. An endoscope is used to check for residual tumor through both the transslyvian and transcallosal routes. Before January 2018, gross total resection (GTR) was assessed using early postoperative MRI. Since January 2018, we have also used intraoperative MRI.

## Results

**Fig. 5:**
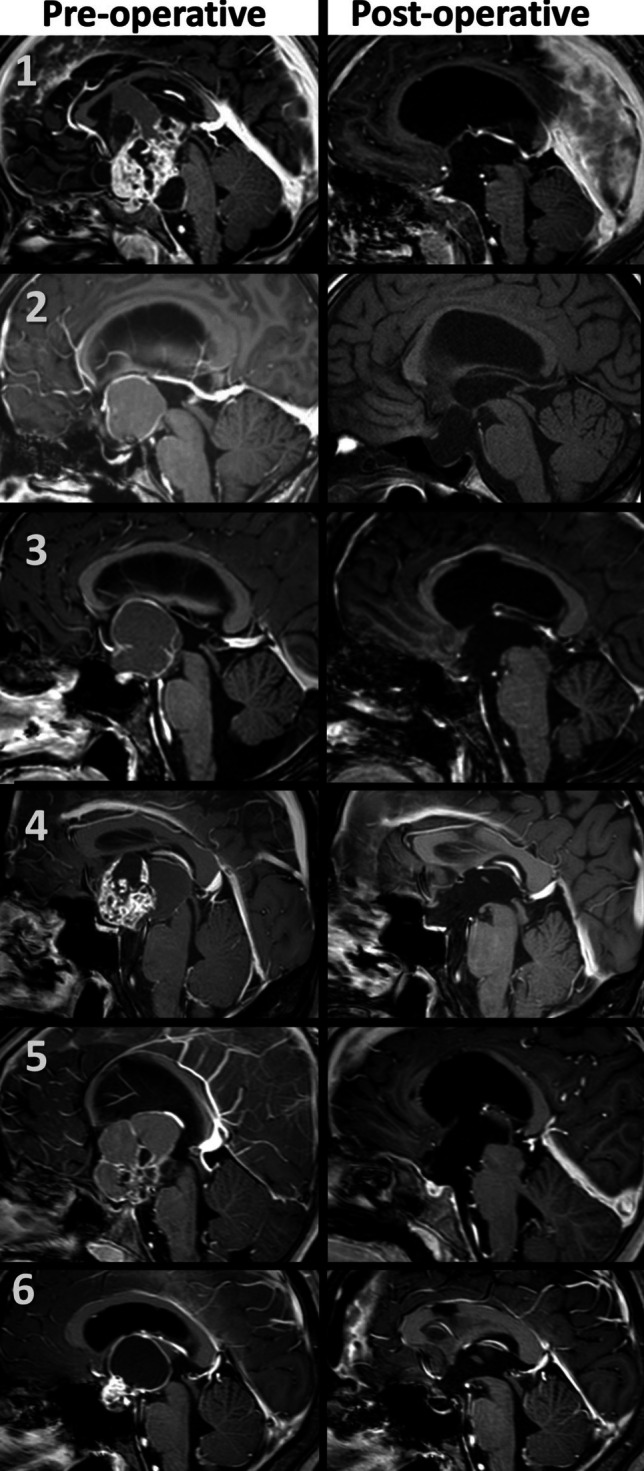
Preoperative and postoperative sagittal contrast-enhanced MRI images of the first 6 patients

**Fig. 6:**
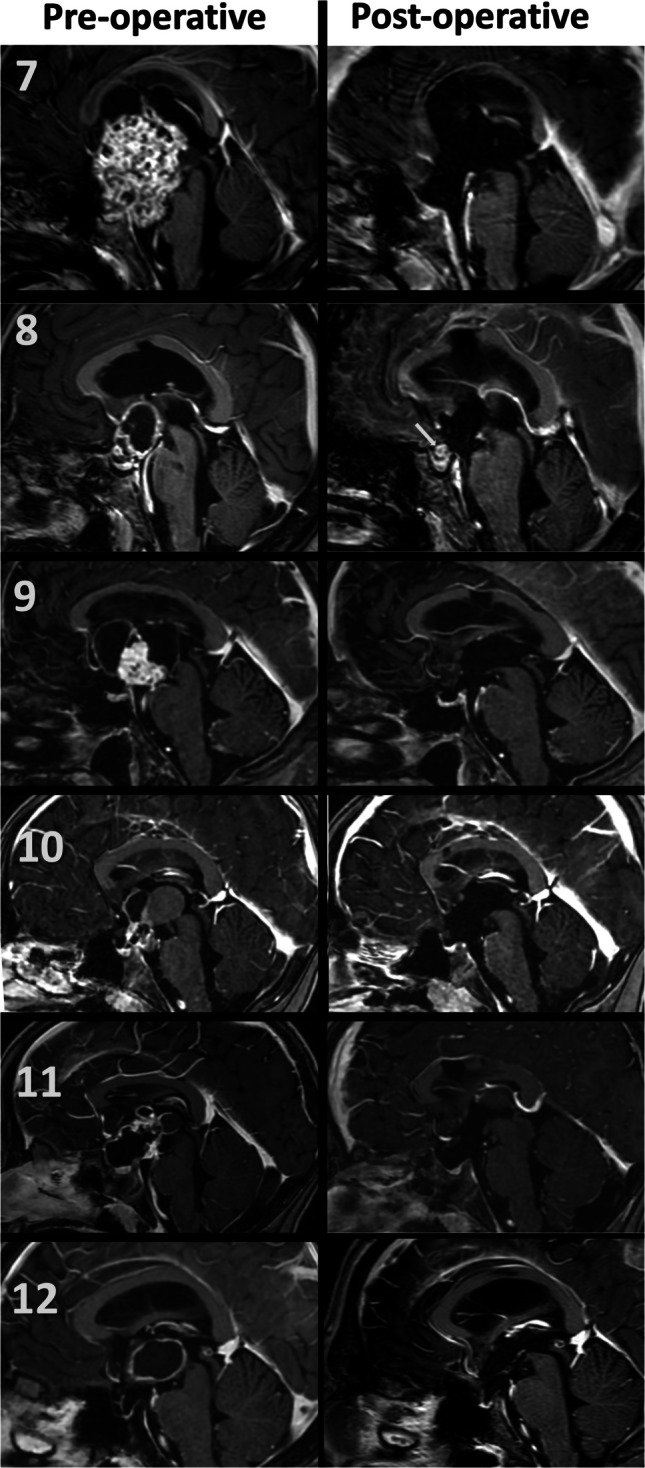
Preoperative and postoperative sagittal contrast-enhanced MRI images of the last 6 patients. The arrow indicates the postoperative recidive tumor of the eighth patient

Since September 2006, 67 patients with a craniopharyngioma have been operated on in our institution by the senior author (UT). Only one patient underwent endonasal transsphenoidal surgery becaue of an intrasellar location. Forty-three patients were operated on through the pterional transslyvian approach for a parachiasmatic location. The combined approach was planned for the remaining 23 patients with intra-extraventricular and pure intraventricular tumors. In 11 of these 23 patients, GTR was achieved only through the AITT approach and no pterional approach was required. In the remaining 12, the operation was combined with a pterional approach because of residual tumor after the AITT approach (Table 1). While primary surgery was carried out in 8 of 12 patients operated on with the combined approach, four underwent surgery because of recidive/recurrent masses. Our GTR rate with the combined approach for intra-extraventricular and pure intraventricular craniopharyngiomas is 91.6% (Figs. 5 and 6). No recurrence was detected in any of the 11 patients who underwent GTR. The only patient experiencing a recurrence during our follow-up had undergone near-total resection and had a history of radiotherapy and two prior surgeries, one at another institution and one at our institution.

**Table 1:** Summary of clinical and surgical data

Case No	Age-Sex	Follow-up (Months)	Symptom	Previous Surgery	Previous RT	Preop. HCP	Preop. DI	Postoperative Hormonal Deficiency & DI	Preoperative Visual Field Test	Postoperative Visual Field Test	Postoperative Complication	Resection Rate	Recurrence	Pathological diagnosis
1	5,F	207	Headache, visual impairment	No	No	Yes	No	Yes	N/A (Pediatric Patient)	N/A (Pediatric Patient)	Postop 6 th month bilateral subdural hematoma (shunt over drainage)	GTR	No	Adamantinomatous craniopharyngioma
2	16,F	205	Menstrual delay, headache	No	No	Yes	No	Yes	Bilateral visual field defects	Improvement	No	GTR	No	Adamantinomatous craniopharyngioma
3	35,F	172	Headache, blurred vision	No	No	Yes	No	Yes	significant bilateral visual field defects	Right homonymous hemianopsia	No	GTR	No	Papillary craniopharyngioma
4	41,M	129	Excessive thirst, forgetfulness, fatigue	No	No	Yes	Yes	Yes	Significant bilateral visual field defects	Bitemporal hemianopsia	No	GTR	No	Adamantinomatous craniopharyngioma
5	6,M	122	Growth in residual tumor	Yes	No	Yes	No	Yes	N/A (Pediatric Patient)	N/A (Pediatric Patient)	No	GTR	No	Adamantinomatous craniopharyngioma
6	19,M	99	Headache	No	No	Yes	No	Yes	Normal	Normal	No	GTR	No	Adamantinomatous craniopharyngioma
7	4,M	97	Oral intake disorder, Sodium imbalance, seizure, drowsiness,	No	No	Yes	Yes	Yes	N/A (Pediatric Patient)	N/A (Pediatric Patient)	Speech disorder	GTR	No	Adamantinomatous craniopharyngioma
8	43,F	88	Blurred vision, fatigue	Yes	Yes	No	No	Yes	Binasal hemianopsia	Binasal hemianopsia	No	NTR	Yes	Adamantinomatous craniopharyngioma
9	52,M	31	Headache	No	No	Yes	No	Yes	Significant bilateral visual field defects	Left Mild Defect (Improved Compared to Pre-op), Right Normal	No	GTR	No	Papillary craniopharyngioma
10	7,F	12	Headache, vomiting, dizziness, excessive thirst, frequent urination, and visual impairment	No	No	No	Yes	Yes	Significant bilateral visual field defects	Left Homonymous Hemianopsia	No	GTR	No	Adamantinomatous craniopharyngioma
11	7,M	7	Severe headache, visual impairment	No	No	No	No	Yes	Bitemporal hemianopsia	Bitemporal hemianopsia	No	GTR	No	Adamantinomatous craniopharyngioma
12	28,M	6	Severe headache, vomiting, visual impairment, growth in residual tumor	Yes	No	Yes	Yes	Yes	Left homonymous hemianopsia	Left homonymous hemianopsia	No	GTR	No	Adamantinomatous craniopharyngioma

*F* Female, *M* Male, *RT* Radiotherapy, *Preop* Preoperative, *DI* Diabetes Insipidus, *GTR* Gross Total Resection, *NTR* Near-total Resection

Among the 12 patients operated on using the combined approach, the last 4 patients, who were operated after January 2018, had the extent of gross total resection (GTR) confirmed by intraoperative MRI, whereas the remaining 8 cases were evaluated with postoperative MRI. Among the remaining 11 cases, in which the transcallosal approach alone was sufficient, intraoperative MRI was used in the last 3 cases, whereas it was not used in the first 8 cases.

The mean follow-up period was 96.9 months (median 98 months, range 5–206), and the mean age was 21.91 years (range 4–52 years). Half of the patients were pediatric, while 7 were male and 5 were female. None of the patients received radiotherapy after our surgery. Postoperative pathology results revealed 10 adamantinomatous craniopharyngiomas and 2 papillary craniopharyngiomas. No mortality was observed in our series, including the follow-up period. Five of six pediatric patients were able to return to school and the adult patients continued with their regular work after surgery.

The tumor stalk was removed in all patients and associated hormonal insufficiency and diabetes insipidus were observed in all patients. All patients received hormone replacement therapy and all pediatric patients were given growth-hormone therapy. After growth-hormone replacement, the patients’ heights reached the average for the population. Furthermore, no mass recurrence associated with growth-hormone usage was identified.

In patient #5, the initial surgery was done at a different center, but resection was subtotal. GTR was achieved with a combined approach during the patient’s second surgery in our institution because of a recurrent mass. Patient #7 of the series was admitted with a severe oral intake disorder. He could be fed only through a nasogastric tube during the radiotherapy planning phase after undergoing an open biopsy and ventriculoperitoneal shunt surgery at another institution. GTR was achieved with a second surgical intervention done at our institution with a combined approach. A speech disorder, which was absent in the early postoperative period, developed in the late postoperative period due to acute hydrocephalus and rapid sodium changes. This patient was also obese and the only one with morbidity. He was the only child receiving special education because of the speech disorder.

Patient #8 had previously undergone subtotal surgical resection followed by radiotherapy at a different institution. In the second operation in our department, resection remained near total. Because of recurrence in the follow-up period, the patient was operated on once again with a combined approach and the tumor was once again near-totally resected. The arachnoid plane between the optic chiasm, anterior communicating artery, and the tumor could not be distinguished because of severe adhesion caused by radiotherapy, making dissection impossible, and residual tumor remained in the chiasm (indicated by an arrow in Fig. 6 on the postoperative image). The tumor recurred 2 years later. The patient’s fourth operation (the third in our institution) was carried out via the pterional approach and the tumor was again excised near-total. This patient is still being closely monitored for recurrence.

Patient #12 underwent surgery 5 years prior to this study at a different institution, where the tumor was subtotally resected through a right pterional approach. The patient experienced progressively worsening headaches and visual impairment, and a recurrent mass was detected. At our institution, the patient was operated on with a combined approach, achieving GTR.

### Illustrative case

**Fig. 7:**
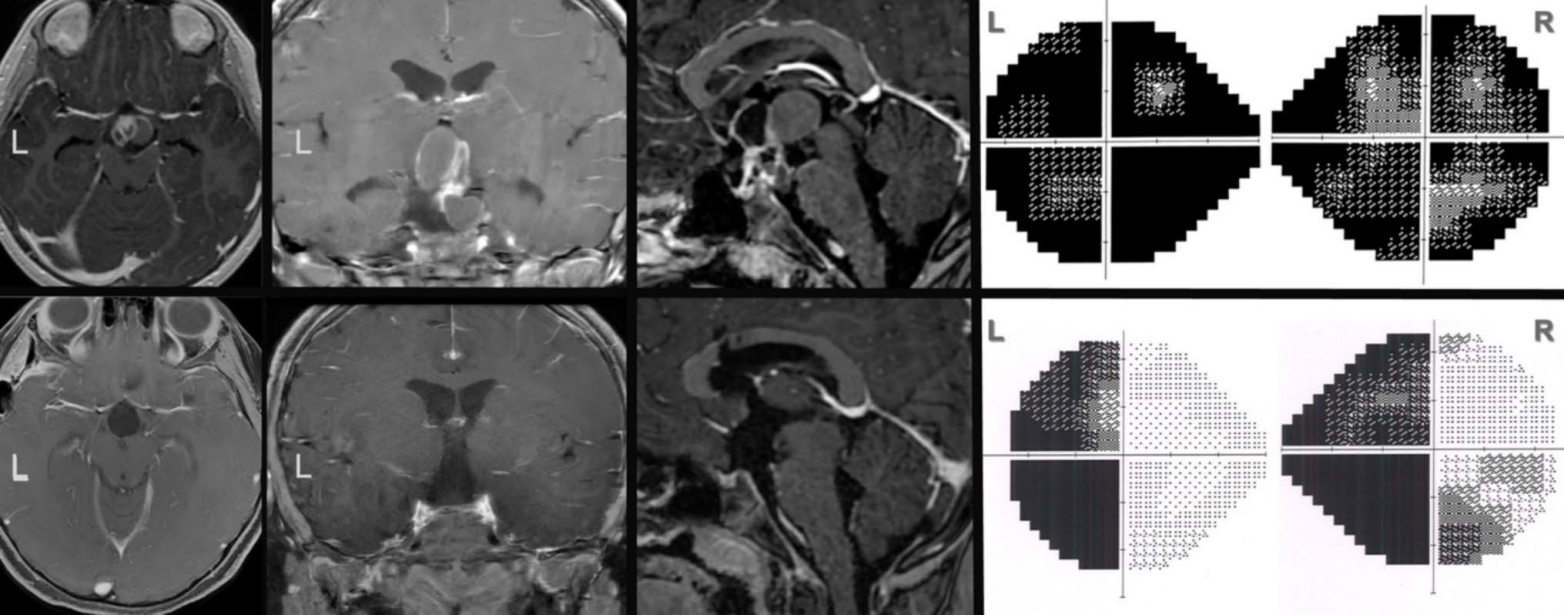
Preoperative and postoperative contrast-enhanced MRI images and visual field tests of the illustrative case (Case 10)

A 7-year-old female presented with complaints of headache, vomiting, dizziness, excessive thirst, frequent urination, and visual impairment. A contrast-enhanced MRI revealed a intra-extraventricular craniopharyngioma. Preoperatively, the patient had mild bilateral lateral ventricle dilation and significant visual field defects. A combined approach for craniopharyngioma resection was planned, and the tumor was gross-totally resected via the interhemispheric transcallosal route combined with a pterional transsylvian route in the same session. As soon as the stalk was cut, desmopressin therapy was initiated. Vision in both of the patient’s eyes improved. With no additional complications, the patient was discharged after a two-week clinical observation. The pathology result showed an adamantinomatous craniopharyngioma. No recurrence was detected during the 11-month follow-up period (Fig. 7, Supplemantary Video 1, Supplemantary Video 2).

## Dıscussıon

One of the appropriate approaches for intra-extraventricular and pure intraventricular craniopharyngiomas was defined by Yaşargil as a combined AITT and pterional approach [7, 31, 38]. In tumors with an intraventricular extension, pulling bluntly from the bottom increases the risk of bleeding because the tumor usually adheres to the choroid plexus and hypothalamus. In the autopsies of two patients who died after craniopharyngioma surgery in the era before computed tomography, Yaşargil found the cause of death to be intraventricular venous hemorrhage [43]. This was the result of hemorrhage from vascular structures in the choroid plexus because the tumor had been blindly pulled from the inferior side. The interhemispheric route should be used for safe and effective resection in patients with intra-extraventricular and pure intraventricular craniopharyngiomas. Although a tumor may provide a clear arachnoid plane with vascular structures during the initial surgery, it often adheres to the hypothalamus. For this reason, adhesions to the hypothalamus should be visualized and dissected through a transcallosal approach.

According to the results of our series of the combined approach, the cornerstone of success in craniopharyngioma surgery is GTR during the first session. In this locally aggressive and relapse-prone tumor, leaving residual tumor is undesirable. In our series, GTR was achieved in all patients who had not undergone previous surgery or radiotherapy, and none of these patients experienced recurrence, even over a long follow-up period. Only one patient experienced recurrence; this patient had undergone near-total resection with prior radiotherapy and surgeries. The possibility of GTR of a residual craniopharyngioma, especially if it was treated with radiotherapy, is very limited in revision surgeries [46]. The tumor’s biology and the effects of radiotherapy make it very difficult to open the adhesion of the tumor to the surrounding neural and vascular structures during revision surgery. Therefore, our basic philosophy for this procedure is total tumor resection in the first surgery, dispelling the need for radiotherapy or revision surgeries [17]. Literature reviews and large series indicate that GTR is superior to radiotherapy after subtotal resection in patients with craniopharyngiomas without hypothalamic involvement and is crucial for achieving low recurrence rates [7, 31, 38].

Our practice is to remove the stalk together with the tumor in all patients to prevent residual tumor. For this reason, diabetes insipidus and pituitary insufficiency were observed in all our patients [1, 9, 25, 32]. However, although less common, stalk-preserving surgery may be considered in selected cases where the tumor origin and growth pattern allow, and the stalk is not invaded. In our series, all tumors were giant craniopharyngiomas with radiological and intraoperative evidence of stalk invasion; therefore, stalk resection was performed in every case [36]. The overall success of surgery depends on close follow-up of the patient by an adult or pediatric endocrinologist in the preoperative and postoperative period and the regulation of hormone replacement therapy and fluid-electrolyte therapy and management of fluid-electrolyte disturbances. Even if there were no additional surgical complications, patients were not discharged for two weeks to allow for close follow-up of endocrinologic complications. To prevent complications from hypernatremia, we initiated intraoperative desmopressin treatment after the stalk was removed. In addition, close nutritional follow-up and family support are extremely important to prevent obesity in these patients. In the treatment of patients with a craniopharyngioma, the multidisciplinary approach involving surgery, intensive care, anesthesia, and endocrinology departments is as crucial as the surgery itself; this constitutes a fundamental component for achieving optimal outcomes.

Especially in pediatric patients, it is advocated to postpone surgery to maintain growth and prevent endocrinological morbidity. Minimally invasive implantation of an Ommaya reservoir is recommended for this group. Treatments such as cyst aspiration through the reservoir and intracyst drug administration have been proposed [15], but numerous reports in the literature describe the neurotoxic effects of these types of local drug applications [6]. Furthermore, our clinical observations suggest that such drugs may stiffen the tumor and cause severe adhesion, thereby complicating excision. Radical surgical removal is well tolerated in the pediatric population with good endocrinologic follow-up and hormone replacement therapy. Total resection is also very important to protect children from the possible side effects of radiotherapy. Radiation exposure in childhood may retard growth, impair brain development, and give rise to secondary cancers [33]. Recently, BRAF inhibitors have been proposed for papillary craniopharyngiomas [24]. Although they are a promising option, more evidence is needed for them to be considered an alternative to GTR. Additionally, these tumors are less commonly seen compared to adamantinomatous craniopharyngiomas. In our series, papillary craniopharyngiomas were observed in only 16% of patients, whereas in a large series from the literature, this rate is only 8% [47].

Ultrasound and endoscopy assistance were routinely used in our cases. Ultrasound is especially used with cottonoids to determine the location of the callosotomy [26]. Residual tumor is checked with an angled endoscope to prevent residual tumor in areas where the microscope is blind. Intraoperative MRI has been routinely used in all our patients since January 2018. However, ultrasound, MRI, and endoscopy may also be insufficient to show residuals at the microlevel and may not completely prevent recurrence [20].

Although a combined approach was planned for 23 patients with hydrocephalus and intraventricular extension in our series, 11 lesions were completely resected only through the AITT approach. For this reason, we prepare the field for the pterional skin incision, but we decide the necessity of the second approach after the transcallosal stage is completed. Preoperatively, it is difficult to predict with certainty whether the transcallosal approach alone will be sufficient. In our series, of the two patients whose tumors were radiologically very similar, one underwent only an AITT approach, while the other required a combined approach. In combined approaches, the necessity of repositioning the head between the transcallosal and pterional stages may raise concerns about sterility. Although it is technically possible to complete the surgery in a single position to avoid this risk of contamination, such setups may compromise optimal surgical angles and ergonomics. In our practice, we prioritize ideal positioning for each corridor and employ a preplanned sterile draping protocol that allows secure transition without compromising the surgical field.

It is imperative that the surgeon have good knowledge of venous anatomy and studies the magnetic resonance venography images on a case-by-case basis. Targeted cleaning of magnetic resonance venographs is necessary to prevent unnecessary complexity. Magnetic resonance venography provides two important facts. The first is the relationship between the coronal suture and superficial cortical veins. The location of the cortical veins is important for the interhemispheric route. Normally, a routine right-sided craniotomy is preferred, but if the cortical veins create a disadvantage on the right side, a left-sided craniotomy may be chosen. The second important fact is the relationship between the foramen of Monro and the junction of the anterior septal vein and thalamostriate vein. Contrary to popular belief, in 50% of hemispheres, the foramen is located on average 6 mm posterior to the venous junction [41]. This distance allows enlargement of the foramen posteriorly. In all patients in this series, the foramen of Monro was sufficiently wide for tumor resection due to hydrocephalus; however, this method can be applied in patients in whom the foramen of Monro is not sufficiently enlarged.

Our combined approach differs from Yaşargil’s in two key aspects: we used separate skin incisions for parasagittal and pterional craniotomies to avoid unnecessarily long incisions, and while we prioritized the transcallosal approach, which obviated the need for the pterional approach in most cases, we initially used the pterional approach in the first two patients.

In the study by Jean WC, the combined approach utilized simultaneous transventricular and orbitofrontal corridors, with the microscope used for the transventricular route and the endoscope for the orbitofrontal route or in vice versa in two cases [23]. Although his technique involved simultaneous use of both corridors, the fundamental surgical philosophy — advocating for the use of multiple pathways to achieve gross total resection in cases where a single corridor would be insufficient — is consistent with the main theme and philosophy of our study.

In addition to the combined approach described by Yaşargil for suprasellar and intraventricular craniopharyngiomas, the transsphenoidal, petrosal, and translaminar approaches have also been described in the literature [3, 8, 10, 14, 16, 19]. In addition to the traditional microsurgical sublabial or endonasal transsphenoidal approach, endoscopic extended transsphenoidal surgery has been used increasingly in recent years [2, 4, 5, 8, 11, 12, 14, 16, 21, 27, 28, 34]. A wider resection opportunity has been provided with the introduction of the endoscope to neurosurgical practice; thus, craniopharyngiomas confined purely to the intrasellar region have become amenable to removal. This is an effective and more minimally invasive approach compared to other approaches in patients with a predominant visual deficit, located only in the sella without suprasellar and intraventricular compartments. However, in large craniopharyngiomas, especially in cases with suprasellar and intraventricular extension, the GTR rate of the transsphenoidal approach drops to a range of 37% to 77% [22, 28, 30, 42].

The indication for the transsphenoidal approach is limited to cases within the sella, as GTR is extremely important in this group of tumors, which are histologically benign but behaviorally very aggressive. Even a very small residual tumor can cause recurrence. Even when combined with radiotherapy or stereotactic radiosurgery, subtotal resection has a higher recurrence risk than GTR [17, 18, 39, 46]. Unfortunately, surgery for a recurrent craniopharyngioma is challenging, with a low chance of GTR [13]. Even in the series reported in the literature with the highest GTR rate with the endonasal approach, residual tumors remain in 23% to 63% of patients [22, 28, 30, 42]. There is no consensus on the management of these residual tumors after transsphenoidal resection. According to the literature and our own experience, treating remaining residual tumors with radiotherapy does not help reduce recurrence; on the contrary, it decreases the chance of GTR in the following surgery [46].

Another question is the issue of CSF leakage after transsphenoidal surgery for large tumors. Craniopharyngioma surgery is a difficult surgery to follow and manage because of hormonal and biochemical imbalances in the early postoperative period, and dealing with CSF leakage in this period creates a handicap for the transsphenoidal approach. In summary, while the transsphenoidal approach may be suitable for cases confined to the sellar region, its use should be avoided for tumors with extensions beyond this area, such as parachiasmatic and intraventricular extensions, because of the high rate of residual tumors, and there is no effective treatment options for these lesions.

The surgery for craniopharyngiomas is extremely challenging, and if gross total resection (GTR) cannot be achieved with an endoscopic endonasal approach, combining this approach with another surgical method should be considered to aim for GTR instead of relying on adjuvant therapies. For instance, combinations such as the endoscopic endonasal-pterional or endoscopic endonasal-transcallosal approaches can be utilized.

A subfrontal translaminar approach is another option for craniopharyngiomas that are limited to the third ventricle [29, 37]. However, this approach is only one of several options within the pterional approach and is already utilized in combined approaches.

Especially in patients with retrochiasmatic giant craniopharyngiomas, the petrosal approach provides access to the portion of the tumor located in front of the midbrain [3, 19]. However, although this approach provides direct access to the inferior and posterior regions of the tumor, the superior part of the lesion remains in the blind spot when the tumor has extended above the foramen of Monro.

According to our clinical experience, the most important issue in craniopharyngioma surgery is total resection during the first surgery. Radiotherapy, stereotactic radiosurgery, or chemotherapy applied to the residual tumor is not as effective as total resection [46]. Carrying out two separate craniotomies in the first operation, as in the combined approach, is much more logical than dealing with a recurrent tumor later on. Unfortunately, recurrence can be seen even in patients having total resection. For this reason, close radiologic follow-up should be done even of patients in whom total resection is accomplished and, if recurrence is detected, revision surgery should be done in the early period before the tumor grows.

## Conclusıon

Craniopharyngiomas are tumors with locally aggressive behavior, a tendency to recur, and a location adjacent to important neurovascular structures. Therefore, it is extremely important to achieve GTR in the initial surgery. Nevertheless, with the combined approach–a combination of the AITT and the pterional transsylvian approaches–tumors in patients with hydrocephalus and intraventricular parachiasmatic extensions have a high rate of GTR, especially in virgin cases. While the AITT and pterional transsylvian combination is effective, surgeons should employ all available surgical techniques, including combined approaches like endoscopic endonasal and pterional approaches, to maximize the chances of achieving gross total resection (GTR), with the specific approach chosen based on surgeon preference and experience. Despite the common practice of administering radiotherapy after subtotal resection, our combined surgical approach demonstrates the possibility of achieving gross total resection without the need for radiation, which importantly preserves the chance for subsequent gross total resection if recurrence or residual tumor occurs.

## Supplementary Information

Below is the link to the electronic supplementary material.

Supplementary file1 (MOV 198552 KB)

Supplementary file2 (MOV 369852 KB)

## Data Availability

All radiological data included study in Figs. 5 and 6.
